# Lactulose Versus Rifaximin Monotherapy for Hepatic Encephalopathy Prevention: A Prospective Comparative Study in Patients With Cirrhosis

**DOI:** 10.7759/cureus.95519

**Published:** 2025-10-27

**Authors:** Areeba Asghar, Awais Mustafa, Jazba Yousaf, Shoukat Hussain, Abdullah Elrefae, Hifza Ishtiaq, Miqdad Qandeel, Maryam Atta, Muhammad Iftikhar Khattak, Aima Tahir

**Affiliations:** 1 Department of Medicine, Russell’s Hall Hospital, The Dudley Group NHS Foundation Trust, Dudley, GBR; 2 Department of Gastroenterology, Faisalabad Teaching Hospital, Faisalabad Medical University, Faisalabad, PAK; 3 Department of Geriatric Medicine, Russell’s Hall Hospital, The Dudley Group NHS Foundation Trust, Dudley, GBR; 4 Department of Endocrinology, Capital Hospital, Islamabad, PAK; 5 Department of Trauma and Orthopedics, AlBashir Hospital, Amman, JOR; 6 Department of Medicine, Abbas Institute of Medical Sciences, Muzaffarabad, PAK; 7 Department of Trauma and Orthopedics, Central Middlesex Hospital, London, GBR; 8 Department of Medicine, Azad Jammu and Kashmir Medical College, Muzaffarabad, PAK; 9 Department of Research and Development, Health Services Academy, Islamabad, PAK; 10 Surgery, Mayo Clinic, Rochester, USA

**Keywords:** cirrhosis, hepatic encephalopathy, lactulose, recurrent he, rifaximin, secondary prophylaxis

## Abstract

Background: Hepatic encephalopathy (HE) is a serious complication of liver cirrhosis, often leading to frequent hospitalizations and reduced quality of life.

Objective: To prospectively compare the efficacy, safety, and tolerability of lactulose versus rifaximin monotherapy in preventing recurrent HE among patients with cirrhosis across multiple tertiary centers.

Methodology: This prospective, multi-center comparative observational study was conducted between November 2023 and October 2024 across three sites: Capital Hospital (Islamabad, Pakistan), AlBashir Hospital (Amman, Jordan), and Abbas Institute of Medical Sciences (Muzaffarabad, Azad Jammu and Kashmir). A total of 236 patients with cirrhosis with a prior episode of overt HE were randomly assigned to receive either rifaximin 550 mg twice daily or lactulose 30 mL three times daily for six months, followed by an additional six months of follow-up. Data were collected on treatment adherence, adverse effects, HE recurrence, and hospitalizations. Statistical analysis was performed using SPSS v26 (IBM Corp., Armonk, NY), with significance set at *P* < 0.05.

Results: Treatment adherence ≥80% was achieved in 102 (86.4%) patients in the rifaximin group versus 85 (72.0%) in the lactulose group (*P* = 0.008). Recurrent HE occurred in 19 (16.1%) rifaximin patients compared to 41 (34.8%) lactulose patients (*P* = 0.001). HE-related hospitalizations were significantly lower in the rifaximin group (15, 12.7%, vs. 36, 30.5%; *P* < 0.001). Adverse effects such as diarrhea (6.8% vs. 27.1%), bloating (5.1% vs. 15.3%), and nausea (3.4% vs. 11.9%) were markedly less frequent with rifaximin. Functional improvement was observed in 94 patients (79.7%) receiving rifaximin compared with 68 patients (57.6%) receiving lactulose (*P* < 0.001).

Conclusions: Rifaximin monotherapy demonstrated superior efficacy, safety, and tolerability compared to lactulose for preventing HE recurrence among patients with cirrhosis in a multi-center setting. These findings support rifaximin as a more effective long-term therapeutic option for secondary prophylaxis of HE.

## Introduction

Hepatic encephalopathy (HE) remains a major and debilitating neuropsychiatric complication of liver cirrhosis, significantly impairing patients’ quality of life and placing a substantial burden on healthcare systems [1-2]. HE develops due to the accumulation of neurotoxins, particularly ammonia, resulting from impaired liver detoxification and alterations in gut microbiota [3]. Clinically, HE can range from subtle behavioral changes to deep coma, often necessitating hospitalization and increasing the risk of morbidity and mortality, especially with recurrent episodes [4].

Secondary prophylaxis of HE is crucial, as patients with a prior episode are at higher risk for recurrence [5]. Current therapeutic strategies primarily aim to reduce intestinal ammonia production and absorption [6]. Lactulose, a non-absorbable disaccharide, remains the standard of care [7]. It acidifies the colonic contents, promotes ammonia excretion, and modulates gut microbiota. However, its efficacy is often limited by poor patient adherence, leading to variable outcomes [8].

Rifaximin, a poorly absorbed broad-spectrum antibiotic, has emerged as a potential alternative or adjunct to lactulose [9]. By targeting ammonia-producing gut bacteria, rifaximin has demonstrated effectiveness in reducing HE recurrence with fewer gastrointestinal side effects [10]. Despite growing interest, the comparative efficacy of rifaximin monotherapy versus lactulose for secondary prophylaxis remains debated, particularly in resource-limited settings where cost and accessibility are critical considerations [11].

Prospective studies comparing these agents in South Asian populations are limited. Given the increasing prevalence of cirrhosis in regions such as Pakistan, identifying the most effective strategy to prevent HE recurrence is essential for reducing hospitalizations and improving patient outcomes. Therefore, this study aimed to evaluate the efficacy and tolerability of lactulose and rifaximin monotherapy in preventing recurrent HE among patients with cirrhosis.

## Materials and methods

Study design and setting

This prospective, observational study with a comparison group involved data collection from three sites: Capital Hospital, Islamabad, Pakistan; AlBashir Hospital, Amman, Jordan; and Abbas Institute of Medical Sciences, Muzaffarabad, Azad Jammu and Kashmir. All study coordination, data management, and statistical analysis were conducted at Abbas Institute of Medical Sciences, which served as the lead center. The study period spanned from November 2023 to October 2024.

Inclusion and exclusion criteria

Patients aged 18 years and older with clinically or radiologically confirmed liver cirrhosis and a documented history of at least one prior episode of overt HE (West Haven grade II or higher) were eligible for inclusion. Only those who were clinically stable and considered suitable for secondary prophylaxis were enrolled. Patients were excluded if they were receiving both lactulose and rifaximin concurrently at baseline; had active gastrointestinal bleeding, hepatocellular carcinoma, renal dysfunction (serum creatinine > 2.0 mg/dL), or severe electrolyte imbalance; or were pregnant or lactating. Additionally, individuals who were non-compliant or unable to complete the follow-up protocol were excluded.

Sample size

A total of 236 patients were enrolled using convenience sampling from the inpatient and outpatient gastroenterology services. The rationale for using convenience sampling was the single-center design and the objective of enrolling all consecutive eligible patients during the defined study period. A formal sample size or power calculation was not performed, as the study was exploratory and intended to reflect real-world clinical practice. However, the final sample size is comparable to that of previously published observational studies evaluating prophylactic treatments for HE, such as those by Paik et al. [12] and Muhammad et al. [13]. This limitation has been acknowledged in the Discussion section.

Dosage and group allocation

A simple randomization method (such as computer-generated random numbers) was used to allocate patients randomly into two treatment groups in a 1:1 ratio. The 118 patients in Group A received rifaximin 550 mg twice daily, the FDA-approved dose for the prevention of recurrent HE. There were also 118 patients in Group B, and they took 30 mL of lactulose three times a day, with the dose adjusted to get two to three soft stools a day. After the final episode of HE, both treatment regimens were continued for six months, and patients were then followed for an additional six months to assess treatment durability and long-term outcomes.

Data collection

Data were collected prospectively using a structured proforma (Appendix) designed by the principal investigator in consultation with senior faculty members. This proforma was utilized at the time of enrollment and during follow-up visits. Baseline demographic and clinical information, including age, gender, cirrhosis etiology, Model for End-Stage Liver Disease (MELD) score, Child-Pugh class, and comorbidities, was recorded for all patients. Clinical details related to previous episodes of HE-such as number, grade (based on West Haven criteria), and time since last occurrence-were also documented. Patients were followed monthly for a total of 12 months (six months on therapy and six months post-therapy). At each follow-up visit, adherence to medication, development of recurrent HE, hospitalizations, adverse events (e.g., diarrhea, abdominal bloating, or nausea), and overall functional status were assessed. HE recurrence was defined as the reappearance of overt neuropsychiatric symptoms requiring clinical intervention and confirmed by treating gastroenterologists using standardized criteria. Data were collected by trained medical personnel and verified by a senior clinician for accuracy and completeness. Efforts were made to ensure patient compliance and minimize loss to follow-up through reminder calls and scheduled clinic appointments. All collected data were anonymized and securely stored for confidentiality and future analysis.

Statistical analysis

We used SPSS version 26 (IBM Corp., Armonk, NY) for the statistical analysis. We showed quantitative values as mean ± standard deviation and categorical variables as frequencies and percentages. We used the independent t-test for continuous variables and the chi-square test for categorical variables to compare the two treatment groups. A *P*-value of less than 0.05 was considered to be statistically significant.

## Results

Table 1 compares the baseline characteristics of 236 patients, equally divided between the rifaximin group (*n* = 118) and the lactulose group (*n* = 118). The mean age was comparable between the groups: 54.72 ± 9.14 years in the rifaximin group and 53.96 ± 8.87 years in the lactulose group (*P* = 0.478). Male patients comprised 80 (67.8%) in the rifaximin group and 78 (66.1%) in the lactulose group. The most common etiology of cirrhosis was hepatitis C, affecting 64 (54.2%) and 61 (51.7%) patients, respectively. Other causes, including hepatitis B, alcohol-related liver disease, NASH, and cryptogenic cirrhosis, did not differ significantly between the groups. The mean MELD scores were 17.25 ± 3.41 and 17.42 ± 3.28 (*P *= 0.667), while Child-Pugh Class B was the most frequent in both groups (55.93% and 57.63%). Comorbid conditions such as diabetes mellitus and hypertension were similarly distributed: 38 (32.20%) and 42 (35.59%) in the rifaximin group versus 36 (30.51%) and 39 (33.05%) in the lactulose group, respectively.

**Table 1:** Baseline demographic and clinical characteristics of patients (n = 236). Independent t-test used for continuous variables; chi-square (χ²) test used for categorical variables; statistical significance at *P* < 0.05.

Variable	Category	Rifaximin group (*n* = 118), *n* (%)	Lactulose Group (n = 118), *n* (%)	Test statistics	*P*-value
Age (years)	Mean ± SD	54.72 ± 9.14	53.96 ± 8.87	t = 0.71	0.478
	18-40 years	24 (20.34)	26 (22.03)	χ² = 0.11	0.739
	41-60 years	68 (57.63)	65 (55.08)	χ² = 0.15	0.701
	>60 years	26 (22.03)	27 (22.88)	χ² = 0.03	0.865
Gender	Male	80 (67.80)	78 (66.10)	χ² = 0.09	0.761
	Female	38 (32.20)	40 (33.90)	-	-
Etiology of cirrhosis	Hepatitis C	64 (54.24)	61 (51.69)	χ² = 0.17	0.684
	Hepatitis B	18 (15.25)	22 (18.64)	χ² = 0.52	0.472
	Alcohol-related	12 (10.17)	10 (8.47)	χ² = 0.22	0.638
	NASH	13 (11.02)	15 (12.71)	χ² = 0.15	0.698
	Cryptogenic/Other	11 (9.32)	10 (8.47)	χ² = 0.06	0.812
MELD score	Mean ± SD	17.25 ± 3.41	17.42 ± 3.28	*t* = -0.43	0.667
Child-Pugh class	Class A	18 (15.25)	20 (16.95)	χ² = 0.14	0.713
	Class B	66 (55.93)	68 (57.63)	χ² = 0.07	0.785
	Class C	34 (28.81)	30 (25.42)	χ² = 0.33	0.566
Comorbidities	Diabetes mellitus	38 (32.20)	36 (30.51)	χ² = 0.08	0.779
	Hypertension	42 (35.59)	39 (33.05)	χ² = 0.15	0.700
	CKD (Stage 1-2)	7 (5.93)	8 (6.78)	χ² = 0.07	0.786

Table 2 outlines prior HE episode details for both groups (*n* = 118 each). The mean number of previous HE episodes was 2.87 ± 1.14 in the rifaximin group and 2.93 ± 1.08 in the lactulose group (*P* = 0.651). The average time since the last HE episode was 1.82 ± 0.62 months and 1.76 ± 0.59 months, respectively (*P* = 0.438). Grade II HE was the most common, occurring in 52 (44.1%) patients in the rifaximin group and 48 (40.7%) patients in the lactulose group. Grade III episodes were reported in 42 (35.6%) and 45 (38.1%) patients, while Grade IV episodes occurred in 24 (20.3%) and 25 (21.2%) patients in the two groups, respectively. No significant differences were noted across these variables.

**Table 2:** Characteristics of previous hepatic encephalopathy episodes. Independent t-test used for continuous variables; chi-square (χ²) test used for categorical variables; statistical significance at *P* < 0.05.

Variable	Category	Rifaximin group (*n* = 118)	Lactulose group (*n* = 118)	Test statistics	*P*-value
Mean number of previous HE episodes	Mean ± SD	2.87 ± 1.14	2.93 ± 1.08	*t* = -0.46	0.651
Most recent HE episode (months ago)	Mean ± SD	1.82 ± 0.62	1.76 ± 0.59	*t* = 0.78	0.438
Grade of most recent HE episode (*n*, %)	Grade II	52 (44.07)	48 (40.68)	χ² = 0.26	0.609
	Grade III	42 (35.59)	45 (38.14)	χ² = 0.14	0.710
	Grade IV	24 (20.34)	25 (21.19)	χ² = 0.03	0.861

Table 3 presents adherence and side effects during the first six months of therapy. High treatment adherence (≥80%) was significantly higher in the rifaximin group, with 102 patients (86.4%) adhering, compared to 85 patients (72.0%) in the lactulose group (*P* = 0.008). Diarrhea was more common in the lactulose group, affecting 32 patients (27.12%) versus 8 (6.78%) in the rifaximin group (*P* < 0.001). Abdominal bloating was reported by 18 (15.3%) lactulose patients and 6 (5.1%) rifaximin patients (*P* = 0.012), while nausea affected 14 (11.9%) and 4 (3.4%) patients, respectively (*P* = 0.015). Therapy discontinuation due to adverse effects occurred in 1 rifaximin patient (0.85%) and 7 lactulose patients (5.93%) (*P* = 0.032).

**Table 3:** Treatment adherence and adverse events during therapy (first six months). Chi-square (χ²) test was used for categorical variables; * indicates statistical significance at *P* < 0.05.

Variable	Category	Rifaximin group (*n* = 118)	Lactulose group (*n* = 118)	Test statistics	*P*-value
Adherence ≥80%, *n* (%)	-	102 (86.44)	85 (72.03)	χ² = 7.09	0.008*
Common adverse effects, *n* (%)	Diarrhea	8 (6.78)	32 (27.12)	χ² = 16.04	<0.001*
	Abdominal bloating	6 (5.08)	18 (15.25)	χ² = 6.34	0.012*
	Nausea	4 (3.39)	14 (11.86)	χ² = 5.87	0.015*
Therapy was discontinued due to side effects	-	1 (0.85)	7 (5.93)	χ² = 4.61	0.032*

Table 4 presents the comparative outcomes after 12 months of follow-up. Patients receiving rifaximin experienced markedly fewer recurrences of HE and hospitalizations than those on lactulose, reflecting a significant clinical advantage. Moreover, the average time to recurrence was notably longer with rifaximin, suggesting more sustained remission. Functional recovery rates were also substantially higher in the rifaximin group, highlighting its therapeutic efficacy in improving overall patient outcomes. Precipitating factors such as gastrointestinal bleeding, dehydration, infection, or electrolyte imbalance were identified in both groups, but their distribution did not differ significantly, indicating that rifaximin’s benefit was consistent regardless of precipitating causes. Loss to follow-up remained low and comparable between the two groups.

**Table 4:** Comparison of clinical outcomes and precipitating factors for recurrent hepatic encephalopathy (HE) between the rifaximin and lactulose groups at 12-month follow-up. Precipitating factors were determined through clinical evaluation and laboratory findings at the time of recurrence. Continuous variables are presented as mean ± standard deviation (SD) and compared using the independent t-test, while categorical variables are expressed as frequency (percentage) and compared using the chi-square (χ²) test. *P < 0.05 was considered statistically significant. HE, hepatic encephalopathy; SBP, spontaneous bacterial peritonitis; UTI, urinary tract infection; SD, standard deviation

Outcome		Rifaximin group (*n* = 118)	Lactulose group (*n* = 118)	Test statistics	*P*-value
Recurrent HE, *n* (%)		19 (16.10)	41 (34.75)	χ² = 10.45	0.001*
HE-related hospitalizations, *n* (%)		15 (12.71)	36 (30.51)	χ² = 11.88	<0.001*
Mean time to HE recurrence (months)		5.62 ± 1.11	4.28 ± 1.39	*t* = 8.18	<0.001*
Overall functional improvement, *n* (%)		94 (79.66)	68 (57.63)	χ² = 14.23	<0.001*
Loss to follow-up, *n* (%)		3 (2.54)	5 (4.24)	χ² = 0.52	0.472
Precipitating factors for recurrent HE, *n* (% of recurrent cases)	Gastrointestinal bleeding	5 (26.3)	8 (19.5)	χ² = 0.39	0.532
	Dehydration/excessive lactulose use	3 (15.8)	9 (22.0)	χ² = 0.29	0.588
	Infection (SBP/UTI/pneumonia)	4 (21.1)	10 (24.4)	χ² = 0.06	0.803
	Electrolyte imbalance	3 (15.8)	6 (14.6)	χ² = 0.01	0.913
	No identifiable precipitant	4 (21.0)	8 (19.5)	χ² = 0.02	0.879

## Discussion

The present study prospectively evaluated the effectiveness of rifaximin plus lactulose compared with lactulose monotherapy in preventing HE recurrence among patients with cirrhosis over a 12-month follow-up period. Both groups were comparable at baseline in terms of demographic and clinical characteristics. The mean age was 54.72 ± 9.14 years in the rifaximin group and 53.96 ± 8.87 years in the lactulose group. Hepatitis C was the predominant etiology of cirrhosis in both groups (54.24% vs. 51.69%), and the mean MELD scores were similar (17.25 vs. 17.42). These demographic features are consistent with previous reports identifying hepatitis C as the leading cause of cirrhosis and the mean patient age around 55 years [14,15]. Importantly, recurrent HE episodes were further analyzed for potential precipitating factors such as gastrointestinal bleeding, dehydration, infection, or electrolyte imbalance. No significant intergroup differences were found, indicating that recurrence rates reflected true treatment effects rather than differences in precipitating events.

Patients demonstrated varying levels of adherence and tolerance to their assigned therapies. Adherence was significantly higher in the rifaximin group (102, 86.44%) compared to the lactulose group (85, 72.03%) (*P *= 0.008). This finding aligns with previous reports, suggesting that patients were more likely to comply with rifaximin therapy due to its superior gastrointestinal tolerability [16]. In contrast, lactulose was associated with a higher incidence of adverse effects such as diarrhea (27.12% vs. 6.78%, *P* < 0.001), bloating (15.25% vs. 5.08%, *P* = 0.012), and nausea (11.86% vs. 3.39%, *P* = 0.015), leading to a greater discontinuation rate (5.93% vs. 0.85%). These observations are consistent with earlier studies [11], which also reported that lactulose commonly causes gastrointestinal discomfort.

After a year, the clinical data showed that rifaximin was more effective. The rifaximin group had a much lower probability of getting HE again (19, 16.10%) than the lactulose group (41, 34.75%) (*P* = 0.001). This rate of recurrence is similar to what was shown before [17], which showed that the lactulose group had a greater risk of recurrence than the rifaximin group. Rifaximin also had a lower hospitalization rate for HE (15, 12.71%) than lactulose (36, 30.51%) (*P* < 0.001). This is comparable to what other studies have discovered [18], which showed that rifaximin greatly lowered the number of hospital readmissions when administered as an extra way to avoid them.

Also, rifaximin had a longer-lasting protective effect (5.62 ± 1.11 months) than lactulose (4.28 ± 1.39 months) (*P *< 0.001). Overall health improvement was reported by 94 (79.7%) patients receiving rifaximin, compared with 68 (57.6%) patients receiving lactulose (*P* < 0.001). This is in line with what other studies found [19], which demonstrated that rifaximin monotherapy improved cognitive function and quality of life.

All of these results contribute to the increasing body of data suggesting rifaximin is better than lactulose at preventing HE from coming back, particularly when quality of life and sticking to therapy are very important. All of this information adds to the growing body of evidence that rifaximin is better than lactulose in preventing recurrent HE, especially when quality of life and sticking to therapy are very important.

Strengths and limitations

A major strength of this study lies in its prospective, multi-center design with a year-long follow-up, allowing for a robust comparison of clinical outcomes, including HE recurrence, hospitalizations, and treatment adherence between rifaximin and lactulose. Data were collected from three collaborating sites, while all study coordination, data management, and statistical analyses were conducted at the lead center (Abbas Institute of Medical Sciences). The relatively large sample size and standardized monitoring improved the reliability and generalizability of the findings within similar populations with cirrhosis. Additionally, stratification of adverse events and detailed adherence tracking provided valuable insights into real-world tolerability. These findings may also contribute to improved future clinical outcomes by optimizing adherence and reducing recurrence rates among patients with hepatic encephalopathy. However, limitations include that the study was conducted within a single regional network, which may restrict external validity across broader geographic or ethnic populations. Moreover, the lack of neuropsychological testing limited the assessment of subclinical cognitive improvements, and potential confounders such as nutritional status and comorbidities were not fully controlled.

## Conclusions

This study demonstrates that rifaximin monotherapy provides significant advantages over lactulose for the secondary prevention of HE in patients with cirrhosis. Patients receiving rifaximin experienced fewer HE recurrences, reduced hospitalization rates, and a longer time to recurrence, collectively indicating improved disease control and enhanced quality of life. Furthermore, rifaximin was better tolerated, with higher treatment adherence and a lower incidence of adverse effects, highlighting its suitability for long-term management. These findings suggest that rifaximin not only improves clinical outcomes but also reduces the overall burden on healthcare resources, making it a more reliable and patient-friendly therapeutic option than lactulose in routine clinical practice.

## Appendices

Appendix 

**Table 5:** Data collection questionnaire — Hepatic Encephalopathy Study

Variable / Question	Response / Entry
Study ID	
Center (site)	☐ Capital Hospital, Islamabad ☐ AlBashir Hospital, Amman ☐ Abbas IMS, Muzaffarabad
Recruitment date (DD/MM/YYYY)	
Inclusion/Exclusion check	☐ Meets inclusion criteria ☐ Exclusion reason: _______
Informed consent obtained	☐ Yes ☐ No (explain)
Patient initials / Identifier	
Date of birth / Age (years)	
Age group	☐ 18–40 ☐ 41–60 ☐ >60
Sex	☐ Male ☐ Female ☐ Other: ___
Etiology of cirrhosis	☐ Hepatitis C ☐ Hepatitis B ☐ Alcohol ☐ NASH ☐ Cryptogenic / Other: ___
MELD score (value)	
Child-Pugh class	☐ A ☐ B ☐ C
Comorbidities (tick all that apply)	☐ Diabetes Mellitus ☐ Hypertension ☐ CKD (specify stage) ☐ Other: ___
CKD stage (if present)	☐ Stage 1 ☐ Stage 2 ☐ Stage 3+ ☐ Not present
Number of prior overt HE episodes	
Time since most recent HE episode (months)	
Grade of most recent HE episode (West Haven)	☐ II ☐ III ☐ IV
Randomization/allocation	☐ Rifaximin ☐ Lactulose
If rifaximin — dose & schedule	Rifaximin 550 mg ___ times/day (enter confirmed dose)
If lactulose — dose & schedule	Lactulose ___ mL ___ times/day (enter prescribed dose / titration)
Date treatment started (DD/MM/YYYY)	
Planned duration of therapy (months)	
Adherence monitoring method	☐ Patient report ☐ Pill count ☐ Caregiver report ☐ Other: ___
Adherence during therapy (first 6 months)	☐ ≥80% ☐ <80% — % (estimate): ___
Adverse effects during therapy (tick & describe)	☐ Diarrhea — onset/date: ___ / severity: ___ ☐ Abdominal bloating — date/severity: ___ ☐ Nausea — date/severity: ___ ☐ Other: ___ (detail)
Therapy discontinued due to side effects	☐ Yes — date: ___ — reason: ___ ☐ No
Any other medications relevant to HE (e.g., antibiotics, lactulose if on rifaximin, etc.)	List medications and dates: ___
Recurrent overt HE during 12-month follow-up	☐ Yes ☐ No
If recurrent HE = Yes — Date of recurrence (DD/MM/YYYY)	
Grade of recurrent HE (West Haven)	☐ II ☐ III ☐ IV
HE-related hospitalization(s)	☐ Yes — number: ___ ☐ No
If hospitalized — Admission date(s) / Discharge date(s)	
Time to first HE recurrence (months)	(calculate or record) ___
Overall functional improvement at 12 months	☐ Yes ☐ No — describe measure/scale used: ___
Loss to follow-up	☐ Yes — date lost: ___ ☐ No
Follow-up visit dates (monthly for 12 months)	1: ___ 2: ___ 3: ___ 4: ___ 5: ___ 6: ___ 7: ___ 8: ___ 9: ___ 10: ___ 11: ___ 12: ___
Any precipitating factors identified during follow-up (e.g., GI bleed, infection, electrolyte disturbance)	☐ Yes — specify: ___ ☐ No
Laboratory values (baseline)	Serum creatinine: ___ mg/dL; (add others if collected) NH3: ___; ALT: ___; AST: ___; INR: ___; Bilirubin: ___
Laboratory values (at recurrence or end-of-study, if available)	Serum creatinine: ___; NH3: ___; INR: ___; Bilirubin: ___; other: ___
Concomitant treatments for cirrhosis/complications during follow-up	(e.g., diuretics, variceal therapy, SBP prophylaxis) list: ___
Investigator assessment of causality for adverse events	☐ Related ☐ Possibly related ☐ Unrelated — comments: ___
Was patient hospitalized for reasons other than HE during follow-up?	☐ Yes — reason/date: ___ ☐ No
Outcome at 12 months / final status	☐ Alive, on therapy ☐ Alive, therapy stopped ☐ Deceased (date & cause): ___
Comments / Remarks	
Data collector name / initials	
Date data entry verified	
Verifier / Senior clinician initials	
Signature (if paper CRF)	
